# Clinicopathologic Features of Early Gastric Cancer after *Heli-cobacter pylori* Eradication in Japanese Patients: Comparative Study between Early (<10 Years) and Late (>10 Years) Onset

**DOI:** 10.3390/cancers16183154

**Published:** 2024-09-14

**Authors:** Hajime Teshima, Takahiro Kotachi, Toshio Kuwai, Akiyoshi Tsuboi, Hidenori Tanaka, Ken Yamashita, Hidehiko Takigawa, Yoshihiro Kishida, Yuji Urabe, Shiro Oka

**Affiliations:** 1 Department of Gastroenterology, Hiroshima University Hospital, Hiroshima 734-8551, Japan; teshima@hiroshima-u.ac.jp (H.T.); kotachi@hiroshima-u.ac.jp (T.K.); atsuboi@hiroshima-u.ac.jp (A.T.); hitanaka@hiroshima-u.ac.jp (H.T.); kenyama@hiroshima-u.ac.jp (K.Y.); hidehiko@hiroshima-u.ac.jp (H.T.); kishida1@hiroshima-u.ac.jp (Y.K.); beyan13@hiroshima-u.ac.jp (Y.U.); oka4683@hiroshima-u.ac.jp (S.O.); 2 Gastrointestinal Endoscopy and Medicine, Hiroshima University Hospital, Hiroshima 734-8551, Japan

**Keywords:** *Heliobacter pylori* eradication, gastric cancer, chronic gastritis, gastric cancer discovered after eradication, late onset vs. early onset

## Abstract

**Simple Summary:**

*Helicobacter pylori* (Hp) eradication is known to prevent gastric cancer development. However, the efficacy of Hp eradication in preventing gastric cancer is limited, and the occurrence of gastric cancer after long-term follow-up post-eradication has been reported in many cases. In recent years, the number of gastric cancer cases detected after eradication has increased, but there is no consensus on the surveillance intervals for long-term follow-up. Although most gastric cancers detected after eradication are differentiated, undifferentiated gastric cancers are also observed. These cancer types differ in their pathological characteristics and malignancy. Therefore, these characteristics need to be considered when conducting surveillance. The purpose of this retrospective study was to characterize post-eradication differentiated and undifferentiated gastric cancers occurring more than 10 years after Hp eradication and to assist in determining appropriate surveillance intervals.

**Abstract:**

Background/Objectives: Eradication therapy for *Helicobacter pylori* gastritis was approved for insurance coverage by the Japanese government in 2013. Since then, the incidence of gastric cancer discovered after eradication (GCAE) has increased. However, there are only a few reports of GCAE diagnosed more than 10 years after eradication. We investigated the clinicopathological characteristics of early-stage GCAE, including histological types and the interval from eradication to diagnosis. Methods: Overall, 379 patients with a total of 448 GCAE lesions treated with endoscopic resection or surgery at our hospital between January 2015 and December 2021 were assessed, and 315 patients with a known interval from eradication to diagnosis of GCAE with a total of 354 lesions were included. We classified the cases into two groups: differentiated-type GCAE (D-GCAE; 279 patients, 318 lesions) and undifferentiated-type GCAE (UD-GCAE; 36 patients, 36 lesions). Results: Smoking and a mild-to-moderate degree of atrophy were risk factors associated with differentiated-type gastric cancer occurring more than 10 years after *H. pylori* eradication. Additionally, the rate of a mixture of histological types with relatively high malignant potential was significantly higher in UD-GCAE presenting more than 10 years after eradication group than those presenting within 10 years after eradication.

## 1. Introduction

*Helicobacter pylori* is the leading cause of gastric cancer. It develops when the bacterium persistently infects the stomach [1,2,3,4]. *H. pylori* infects approximately 44.3% of all adults worldwide [5]. It is strongly associated with gastric cancer, accounting for about 89% of cases [6]. The prevalence of H. pylori infection is expected to decrease owing to economic development, improved hygienic conditions, and the implementation of eradication therapy, which may also contribute to a reduction in the number of patients with gastric cancer.

*H. pylori* eradication therapy significantly reduces the incidence of gastric cancer [7,8,9,10,11]; however, it cannot completely prevent the occurrence of gastric cancer after eradication (GCAE). The risk of gastric cancer persists even after *H. pylori* eradication because of residual gastric mucosal damage and pre-existing atrophic changes [12].

In Japan, eradication therapy for chronic gastritis associated with *H. pylori* infection was approved for insurance coverage more than 10 years ago, and the same concern regarding the occurrence of GCAE has arisen in these cases. It is generally believed that it takes approximately 10 years for gastric cancer to grow to a size that can be detected [8]. Therefore, a GCAE detected within a few years of eradication may potentially have been present at the time of eradication, whereas a GCAE detected long after eradication (more than 10 years) could be considered a “true” GCAE. Thus, the characteristics of the GCAE are expected to vary depending on the number of years since eradication and the condition of the gastric mucosa. However, there are few reports on the characteristics of long-term post-eradication GCAE. In this study, we investigated the clinicopathological characteristics of early-stage GCAE, including histological types and interval from eradication, and compared those of GCAE presenting within and >10 years after eradication to reveal the clinicopathological characteristics of true GCAE and appropriate post-*H. pylori* eradication therapy surveillance, especially for patients with chronic gastritis. This study also aims to contribute to the development of appropriate surveillance strategies for gastric cancer prevention.

## 2. Materials and Methods

### 2.1. Study Design

This single-center, retrospective, observational cohort study was conducted at the Hiroshima University Hospital. This study was conducted in accordance with the principles of the Declaration of Helsinki. Written informed consent for the procedure was obtained from all the patients. The study protocol was approved by the Ethics Committee of Hiroshima University Hospital (approval number: E2023-0213).

### 2.2. Patients

**Figure 1:**
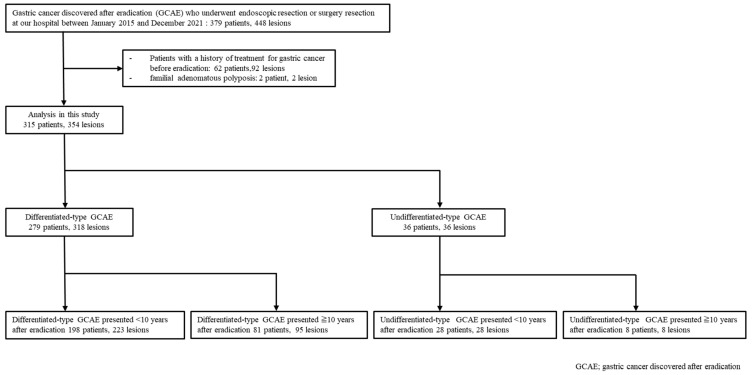
Flowchart showing data of patients enrolled in this study.

We retrospectively assessed 379 patients with 448 GCAE lesions who underwent endoscopic resection or surgery at the Hiroshima University Hospital between January 2015 and December 2021. Among these cases, 62 patients with 92 lesions who had undergone gastric cancer treatment before eradication and two patients with familial adenomatous polyposis (FAP) were excluded from the study. Finally, 315 early gastric cancer (EGC) cases with 354 lesions (230 males and 85 females; mean age, 70 years) were included in this study (Figure 1). The median follow-up was 5.2 years (range: 2.4–11.9 years). The patients were divided into two groups: Differentiated-type GCAE (D-GCAE: 279 patients, 318 lesions) and undifferentiated-type GCAE (UD-GCAE: 36 patients, 36 lesions). For D-GCAE, we compared the characteristics of GCAE that presented within 10 years after eradication (198 patients, 223 lesions) with those that presented >10 years (81 patients, 95 lesions). For UD-GCAE, we also compared the characteristics of GCAE that presented within 10 years of eradication (28 patients, 28 lesions) with those that presented >10 years (8 patients, 8 lesions; Figure 1).

### 2.3. Data Collection and Definitions

The following clinicopathological characteristics were evaluated: patient demographics (age, sex, smoking history, and family history of gastric cancer), endoscopic findings (degree of atrophy, macroscopic type, location, and color tone), and histopathological characteristics (tumor size, depth of invasion, and lymphovascular invasion). The *H. pylori* eradication group was defined as follows: a known time of eradication and *H. pylori*-negative status confirmed by a stool antigen test (Meridian Inc., Cincinnati, OH, USA) or a ^13^C-urea breath test (Otsuka Pharmaceutical Co., Ltd., Tokushima, Japan) [13]. The tumor site and gross type of gastric tumor were classified according to the Japanese Classification of Gastric Cancer (JCGC) [14]. In this study, type 0-I (protruding) and type 0-IIa (superficial elevated) were described as “elevated”, while type 0-IIc (superficial depressed) and type 0-IIa + IIc (elevated with central depression) were described as “depressed”. Endoscopic evaluation of atrophic gastritis was performed according to the Kimura and Takemoto classification criteria [13]. The pathological diagnosis of each tumor was made according to the JCGC criteria [14]. Smoking was defined as regular consumption of at least five cigarettes per day.

### 2.4. Statistical Analysis

Data are expressed as mean ± standard deviation. Fisher’s exact test was used to compare qualitative variables, and the Wilcoxon rank-sum test was used to compare quantitative variables. We evaluated these associations using a multiple logistic regression analysis. The odds ratios (OR) and 95% confidence intervals (95% Cl) were calculated, and values with *p* < 0.05 were considered statistically significant. All data were statistically analyzed using JMP statistical software. 16.2.0 (SAS Institute, Cary, NC, USA).

## 3. Results

### 3.1. Clinical Characteristics of the Patients and Lesions

The characteristics of patients in the D-GCAE and UD-GCAE groups are shown in Table 1. In the D-GCAE group, the mean age was 71 years, and 76% of the patients were male. The mean tumor size was 10 mm. The smoking history rates were 62%, and the mean eradication period was 5 years. Of 279 patients, 20 (8%), 111 (38%), and 148 (54%) patients had mild, moderate, and severe gastric mucosal atrophy, respectively. Of 318 lesions, 38 (12%), 147 (46%), and 133 (42%) lesions developed in the upper, middle, and lower third of the stomach, respectively. The 223 lesions (70%) were diagnosed as the depressed type, while the others were of the elevated type. The 287 lesions (90%) were diagnosed as mucosal invasion depth, while the others were submucosal invasion depth. The 32 patients (12%) had synchronous tumors.

In the UD-GCAE group, the mean age was 67 years, and 52% of the patients were male. The mean tumor size was 15 mm. The smoking history rate was 44%, and the mean eradication period was 4 years. Of 236 patients, 5 (14%), 20 (56%), and 11 (40%) patients had mild, moderate, and severe gastric mucosal atrophy, respectively. Of 36 lesions, five (14%), 21 (56%), and 10 (28%) lesions developed in the upper, middle, and lower thirds of the stomach, respectively. The 32 lesions (89%) were diagnosed as being the depressed type, and the others as the elevated type. The 24 lesions (67%) were diagnosed as mucosal invasion depth, while the others were submucosal invasion depth. Two patients (6%) had synchronous tumors.

### 3.2. Comparison of D-GCAE Presented within and >10 Years after Eradication

In the D-GCAE group, we compared the characteristics of GCAE that presented within 10 years of eradication (198 patients, 223 lesions) and GCAE that presented more than 10 years after eradication (81 patients, 95 lesions; Table 2). Univariate analysis showed a significant difference in smoking history (with/without 111/88 vs. 57/26, *p* = 0.044), gastric mucosal atrophy severity ratio (144, 57.0% vs. 10, 34.5%), and tumor location (U, M/L: 140/83 vs. 45/50, *p* = 0.011) between GCAE presented within and >10 years after eradication.

Multivariate analysis showed that smoking (odds ratio [OR], 2.17; 95% confidence interval [CI], 1.11–4.22; *p* = 0.045) and mild-to-moderate gastric mucosal atrophy (OR, 1.82; 95% CI, 1.04–3.18; *p* = 0.036) were significant independent risk factors for the >10 years after eradication group. Gastric mucosal atrophy was more frequently diagnosed as mild-to-moderate >10 years after eradication than within 10 years of eradication (OR: 1.82, 95% confidence interval [CI], 1.04–3.18; *p* = 0.036). There were no significant differences in age, family history of gastric cancer, tumor size, macroscopic type, treatment, depth of invasion, or synchronous tumors.

### 3.3. Comparison of UD-GCAE Presented within and over 10 Years after Eradication

In the UD-GCAE group, we also compared the characteristics of GCAE presented within 10 years after eradication (28 patients, 28 lesions) and GCAE presented >10 years after eradication in eight patients and eight lesions (Table 3). Univariate analysis showed a difference in histological type (differentiated mixed-type/pure undifferentiated-type: 3/25 vs. 5/3, *p* < 0.01) between GCAE presenting within 10 years after eradication and GCAE presenting >10 years after eradication.

## 4. Discussion

In this study, we revealed differences in clinicopathological features between GCAE presenting within and >10 years after eradication, showing that the rates of smoking history and mild-to-moderate gastric mucosal atrophy were significantly higher in the D-GCAE presented >10 years after eradication group than those within 10 years group. In addition, the rate of a mixture of histological types was significantly higher in the UD-GCAE presented >10 years after eradication group than in the group 10 years after eradication.

For D-GCAE, multivariate analysis showed that smoking history and mild-to-moderate gastric mucosal atrophy were significant independent risk factors for the >10 years after eradication group compared to those within 10 years. Previous studies have reported that the occurrence of GCAE is influenced by several factors including age, time since eradication, smoking history, and severe gastric mucosal atrophy [1,2,3,7,15,16]. Most patients with GCAE present with differentiated-type histology, which could be considered consistent with the characteristics of D-GCAE [17,18,19] and is consistent with our findings regarding smoking history, but different from our findings regarding gastric mucosal atrophy. In addition, the risk of intestinal-type gastric cancer did not change between the first and second decades of follow-up, regardless of the grade of baseline gastric mucosal atrophy [9,20,21,22]. One possible reason for the difference between their results and ours is that we excluded patients with a history of gastric cancer treatment before eradication and included many cases of eradication due to chronic gastritis. Patients with a history of gastric cancer treatment tend to have more severe gastric mucosal atrophy. Mucosal atrophy takes a long time to improve after Hp eradication [23,24], and moderate-to-severe atrophy or intestinal metaplasia may represent a “point of no return” Refs. [25,26]. It was important to exclude patients who had previously been treated for gastric cancer to match the background of the study participants with those receiving eradication therapy for chronic gastritis, a mucosal condition that is expected to improve in the future. Since most patients who will be eradicated will have no history of gastric cancer and will only have chronic gastritis in the near future, our data must be more relevant. Based on these results, it could be recommended that long-term surveillance esophagogastroduodenoscopy (EGD) after Hp eradication is necessary, even in cases of mild-to-moderate gastric mucosal atrophy. In addition, smoking cessation counseling is crucial.

Regarding UD-GCAE, the rate of a mixture of histological types was significantly higher in UD-GCAE patients who presented >10 years after eradication than in those who presented within 10 years (differentiated mixed-type/pure undifferentiated-type: 3/25 vs. 5/3, *p* < 0.01). Patients with undifferentiated gastric cancer often present at advanced stages, leading to rapid progression and worse outcomes than those with differentiated gastric cancer [27,28] However, the number of cases of undifferentiated gastric cancer detected after *H. pylori* eradication is small, and its characteristics remain unclear [9,20,21,22,29]. There is a higher incidence of UD-GCAE in patients with mild atrophy detected >10 years after eradication than in those with mild atrophy detected within 10 years of eradication [1,2,15]. Meanwhile, Kodama et al. reported that the incidence of UD-GCAE remained unchanged over a longer period after eradication [30]. In this study, there were no differences in these variables of UD-GCAE between those presented within and >10 years after eradication. However, the percentage of cases with differentiated mixed-type cancer was significantly higher than that of cases with pure undifferentiated-type cancer by focusing on the primary and secondary histological types (comparing the rates of pure undifferentiated-type and differentiated mixed-type cancers). The process of long-term repair of gastric mucosal atrophy is thought to promote the development of mixed cancers [31]. A meta-analysis shows that mixed undifferentiated-type gastric cancer has more malignant potential than pure undifferentiated-type gastric cancer [32]. These findings suggest that GCAE has a high proportion of histological types (mixed differentiated/pure undifferentiated) more than 10 years after eradication, which may lead to the development of tumors with relatively high malignant potential.

Regarding surveillance, frequent surveillance following *H. pylori* eradication has been reported to be effective in the early detection of EGC Ref. [33]. Long-term endoscopic follow-up of patients cured of *H. pylori* infection revealed that the risk of GCAE was greater at more than 10 years of follow-up than within 10 years [9,20,21,22]. Based on the results of this study, and in conjunction with earlier reports, close annual surveillance is recommended for a prolonged period, even more than 10 years, after eradication.

This study had several limitations. First, this was a single-center, retrospective study. Second, the sample size of patients with undifferentiated-type cancer was small, and only univariate analysis could be performed. Third, there was insufficient information regarding the reason for eradication in each case, although patients with a history of treatment for gastric cancer before eradication were excluded. Fourth, the issue of surveillance intervals for each case is clarified. Fifth, this study does not include data on gastric cancer cases without *H. pylori* infection, which limits the ability to directly compare GCAE with uninfected gastric cancer. To clarify this issue, a large-scale prospective cohort study stratified by *H. pylori* infection status is essential.

Although our study included the largest sample size among the previous studies, it is important to conduct multicenter studies in the future to accumulate and investigate more cases. Moreover, molecular and genetic analyses of post-eradication gastric tissues have not yet been sufficiently conducted to determine how the period following *H. pylori* eradication may influence the key characteristics of gastric tumors. Therefore, a large-scale prospective cohort study, stratified by *H. pylori* infection status, is essential to further advance our understanding in this area.

## 5. Conclusions

In conclusion, smoking and a mild-to-moderate degree of atrophy were risk factors associated with differentiated-type gastric cancer occurring more than 10 years after *H. pylori* eradication. Additionally, the rate of a mixture of histological types with relatively high malignant potential was significantly higher in cases of UD-GCAE that presented more than 10 years after eradication than in those that presented within 10 years after eradication. Therefore, annual surveillance EGD is recommended for a prolonged duration (more than 10 years after *H. pylori* eradication), even in cases of mild-to-moderate gastric mucosal atrophy.

## Figures and Tables

**Table 1:** Comparison of differentiated-type and undifferentiated-type gastric cancer after eradication (GCAE).

Baseline Characteristics	Differentiated-Type GCAE	Undifferentiated-Type GCAE	Total
	(*n* = 279 Patients, 318 Lesions)	(*n* = 36 Patients, 36 Lesions)	
Mean age, y. o. [range]	71 (40–86)	67 (39–87)	70 (39–87)
Sex, male	211 (76)	19 (52)	230 (73.0)
Mean tumor size, mm. [range]	10 (2–90)	15 (3–70)	10 (2–90)
Smoking	172 (62)	16 (44)	188 (60)
Gastric cancer family history, n (%)	82 (29)	13 (26)	95 (30)
Mean eradication period	5 (1–27)	4 (1–30)	5 (1–30)
Gastric mucosal atrophy			
Mild	20 (8)	5 (14)	25 (8)
Moderate	111 (38)	20 (56)	131 (41)
Severe	148 (54)	11 (40)	159 (51)
Location			
Upper third	38 (12)	5 (14)	43 (12)
Middle third	147 (46)	21 (58)	168 (47)
Lower third	133 (42)	10 (28)	143 (41)
Macroscopic type			
Superficial elevated	95 (30)	4 (11)	99 (28)
Superficial depressed	223 (70)	32 (89)	255 (72)
Treatment			
Endoscopic resection	293 (92)	19 (53)	312 (88)
Surgical resection	25 (8)	17 (47)	42 (12)
Invasion depth			
Mucosa	287 (90)	24 (67)	311 (88)
Submucosa	31 (10)	12 (33)	43 (12)
Synchronous tumor			
Positive	32 (12)	2 (6)	34 (78)
Negative	247 (88)	34 (94)	281 (22)

GCAE: gastric cancer discovered after eradication (%).

**Table 2:** Univariate and multivariate analysis for differentiated-type GCAE presented ≥10 years after eradication.

Variables	Eradication Period (Years)		Univariate Analysis	Multivariate Analysis	
	<10, 198 Patients 223 Lesions	≥10, 81 Patients 95 Lesions	*p*-Value	OR (95% CI)	*p*-Value
Age (years)			0.250	1.43 (0.81–2.52)	0.208
<70	94 (47)	33 (40)			
≥70	104 (53)	48 (60)			
Sex			0.796	1.18 (0.56–2.49)	0.613
Male	149 (75)	62 (77)			
Female	49 (25)	19 (23)			
Smoking	114 (57)	58 (71)	0.033	2.17 (1.11–4.22)	0.023
Gastric cancer family history	59 (29)	23 (28)	0.788	0.96 (0.58–1.61)	0.862
Gastric mucosal atrophy			0.031	1.82 (1.04–3.18)	0.036
Mild/moderate	85 (43)	46 (57)			
Severe	113 (57)	35 (43)			
Tumor size (mm)			0.414	0.82 (0.58–1.35)	0.709
≤10	118 (52)	55 (58)			
>10	105 (48)	40 (42)			
Tumor location			0.011	1.53 (0.87–2.69)	0.135
Upper/middle	140 (63)	45 (47)			
Lower	83 (37)	50 (53)			
Macroscopic type			0.136	0.73 (0.42–1.23)	0.759
Superficial elevated	61 (27)	34 (36)			
Superficial depressed	162 (73)	61 (64)			
Treatment			0.261	1.38 (0.42–4.54)	0.590
Endoscopic resection	203 (91)	90 (95)			
Surgical resection	20 (9)	5 (5)			
Invasion depth			0.178	1.41 (0.44–4.47)	0.557
Mucosa	198 (89)	89 (94)			
Submucosa	25 (11)	6 (6)			
Synchronous tumor			0.410	0.77 (0.42–1.38)	0.588
Positive	21 (11)	11 (15)			
Negative	177 (89)	70 (85)			

**Table 3:** Univariate analysis for undifferentiated-type GCAE presented ≥10 years after eradication.

Variables	Eradication Period (Years)		*p*-Value
	<10, 28 Patients, 28 Lesions ≥10, Eight Patients, Eight Lesions		
Age (years)			0.485
<70	21 (75)	5 (63)	
≥70	7 (25)	3 (37)	
Sex			0.858
Male	15 (54)	4 (50)	
Female	13 (46)	4 (50)	
Smoking	13 (46)	3 (37)	0.654
Gastric cancer family history	9 (32)	4 (50)	0.360
Gastric mucosal atrophy			0.698
Mild/Moderate	19 (68)	6 (75)	
Severe	9 (32)	2 (25)	
Tumor size (mm)			0.120
≤10	9 (32)	5 (63)	
>10	19 (68)	3 (37)	
Tumor location			0.274
Upper/Middle	19 (68)	7 (88)	
Lower	9 (32)	1 (12)	
Macroscopic type			0.887
Superficial elevated	3 (11)	1 (13)	
Superficial depressed	25 (89)	7 (87)	
Histological type			<0.01
Differentiated mixed type	3 (11)	5 (63)	
Pure undifferentiated-type	25 (89)	3 (37)	
Treatment			0.326
Endoscopic resection	16 (57)	3 (38)	
Surgical resection	12 (43)	5 (62)	
Invasion depth			0.776
Mucosa	19 (68)	5 (63)	
Submucosa	9 (32)	3 (37)	
Synchronous tumor			0.376
Positive	1 (3)	1 (12)	
Negative	27 (97)	7 (88)	

## Data Availability

The data that support the findings of this study are available from the corresponding author upon reasonable request.
